# Orbital Abscess—Two Case Reports with Review

**DOI:** 10.1007/s12070-021-02486-z

**Published:** 2021-03-05

**Authors:** Tomasz Zawadzki, Oskar Komisarek, Jacek Pawłowski, Bartosz Wojtera, Joanna Bilska-Stokłosa, Krzysztof Osmola

**Affiliations:** 1 Chair and Clinic of Maxillofacial Surgery, Poznan University of Medical Sciences, Poznan, Poland; 2 Department of Maxillofacial Orthopaedics and Orthodontics, Poznan University of Medical Sciences, 60- 356 Poznan, Poland; 3 Students Research Group of Chair and Clinic of Maxillofacial Orthopedics and Orthodontics, Poznan University of Medical Sciences, Poznan, Poland

**Keywords:** Orbital abscess, Orbital trauma, Treatment

## Abstract

Periorbital infections lead to severe condition of the orbital abscess, and eventually to sight loss, and even death. Current study aims in reviewing the literature regarding orbital abscess in adult patients and presenting 2 original cases. A surgical intervention to drain the abscess and a revision of the orbital was required. A review of literature is also reported focusing on aetiology and treatment options dealing with an orbital abscess.

## Introduction

Periorbital infections lead to severe condition of orbital abscess, and eventually to sight loss, and even death [1, 2]. They carry the risk of rapid deterioration, hence require immediate management [3].

In 1970, Chandler et al. proposed the classification of orbital complications depending on its extention: I—preseptal cellulitis; II—orbital cellulitis; III—subperiosteal abscess; IV—orbital abscess; V—cavernous sinus thrombosis [4]. Current study aims in reviewing literature regarding orbital abscess in adult patients and presenting 2 original cases.

### Case Report 1

**Fig. 1:**
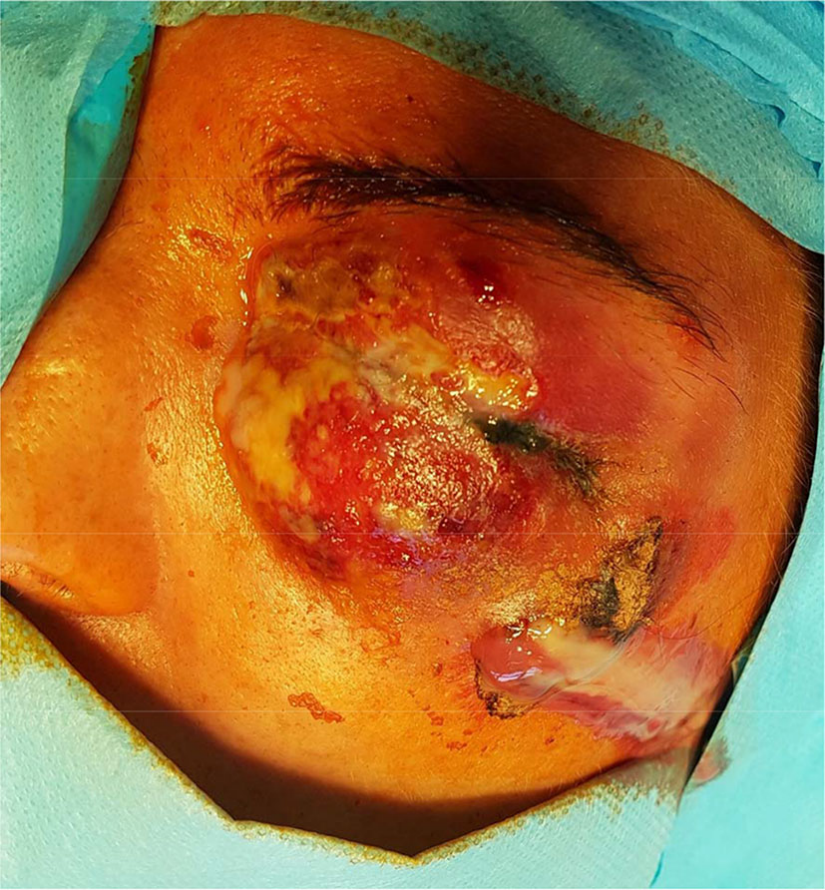
The physical examination shows massive swelling of the eyelids of the left eye—closing the eyelid gap, exophthalmos of the left eyeball, severe pain on palpation, redness and warming of the surrounding soft tissues, eruptions on the skin of the upper and lower eyelids

**Fig. 2:**
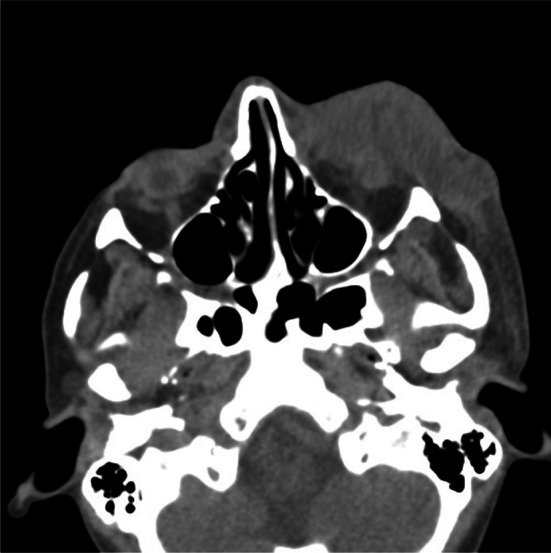
The computed tomography image shows phlegmon of the left cheek and orbital

**Fig. 3:**
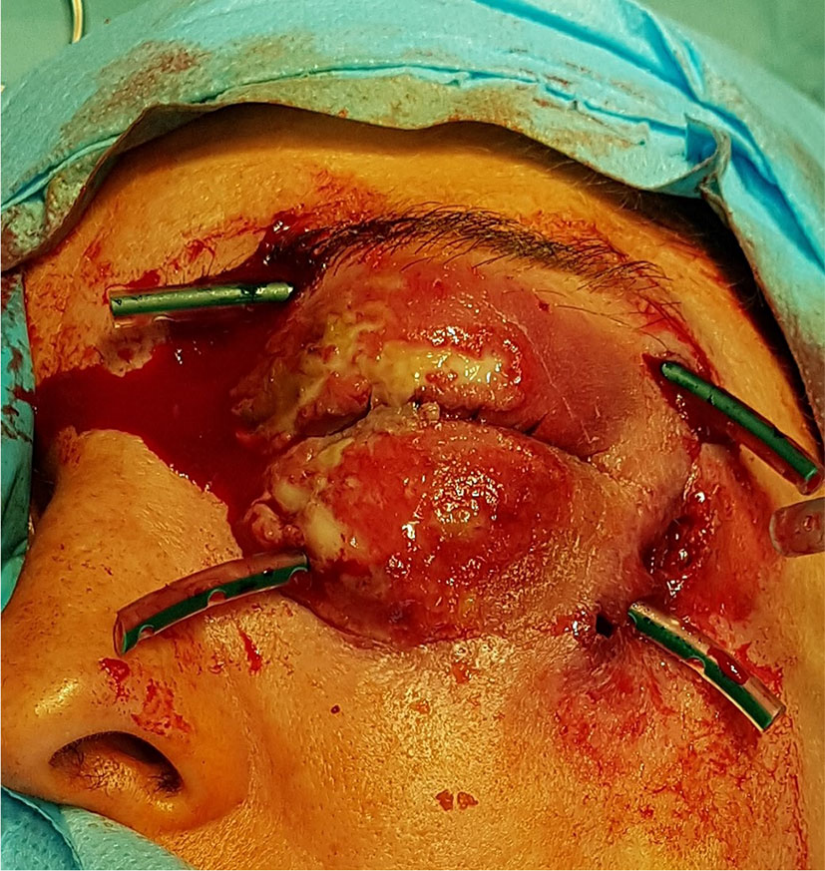
An incision and drainage of the left orbital phlegmon were performed from the supraorbital and suborbital incisions, resulting in abundant purulent exudate

**Fig. 4:**
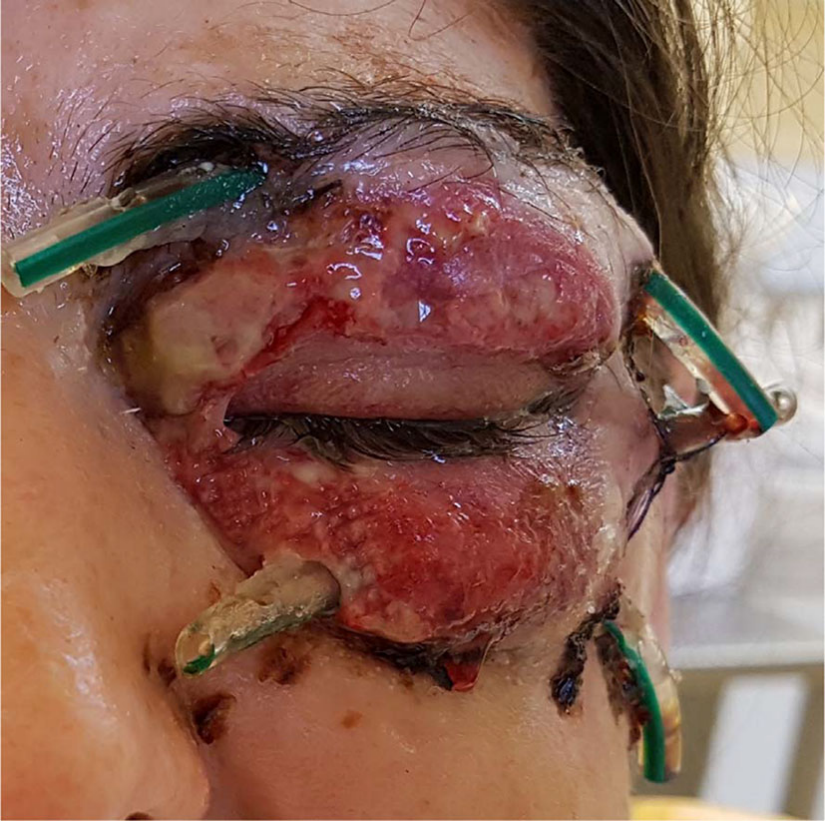
On the third day after surgery, a control ophthalmological examination confirmed correct vision in the left eye

**Fig. 5:**
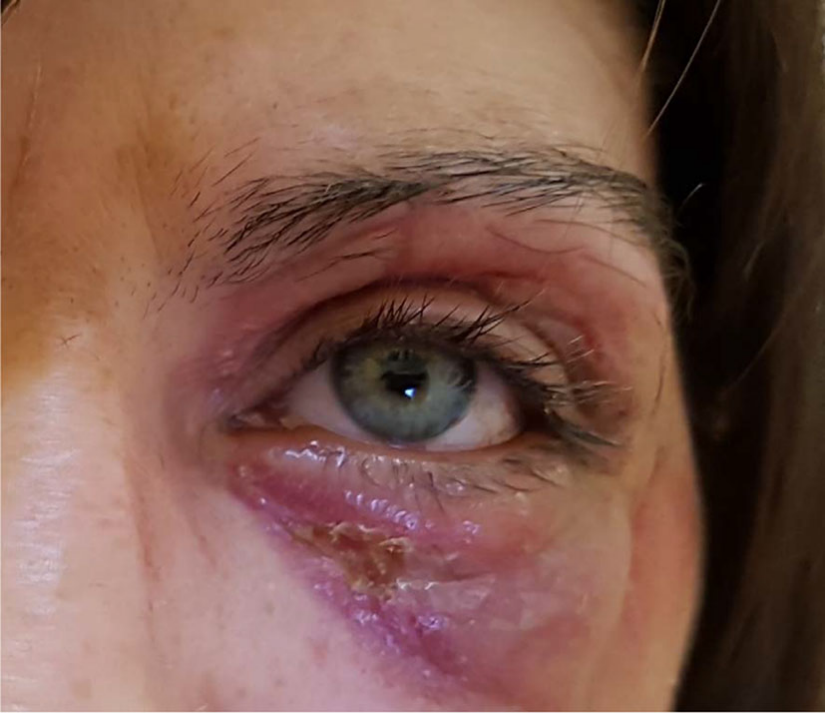
The only permanent consequence was scarring of the facial skin after surgical access

A 35-year-old woman presented to the maxillofacial surgery department in Poznań due to massive eyelid swelling and severe pain in the left eye. Three days before the patient was admitted to the department, she was injured with a blunt instrument. The physical examination shows massive swelling of the eyelids of the left eye—closing the eyelid gap, exophthalmos of the left eyeball, severe pain on palpation, redness and warming of the surrounding soft tissues, eruptions on the skin of the upper and lower eyelids, body temperature 37.9 °C (Fig. 1). No other irregularities were found. Computed tomography of the orbital without contrast and an X-ray of the lungs, laboratory tests, electrocardiogram were ordered. Additional a smear was taken for bacteriological examination. The computed tomography image shows phlegmon of the left cheek and orbital (Fig. 2). In the ophthalmological examination, the right eye remained unchanged. In the left eye, there was an abscess of the eyelids and orbit, swelling of the eyeball and eyelid conjunctiva; transparent cornea; iris unchanged; the pupil is even, round and reacts correctly to light. The image of the fundus of the right eye was normal, the left eye was not available for examination. The patient was administered amoxicillin and clavulanic acid 1.2 g intravenous (IV) three times a day, Metronidazole 500 mg three times daily IV, ketoprofen 0.1 g twice daily, enoxaparin 0.4 ml once daily subcutaneous. Additionally, drops containing dexamethasone and tobramycin every two hours were used for the left eye. Under general endotracheal anaesthesia, an incision and drainage of the left orbital phlegmon were performed from the supraorbital and suborbital incisions, resulting in abundant purulent exudate (Fig. 3). The abscess cavity was rinsed with saline. A flow drain was introduced. In the postoperative period, the level of CRP and WBC was monitored—a decrease in CRP and WBC was observed. On the third day after surgery, a control ophthalmological examination confirmed correct vision in the left eye (Fig. 4). The microbiological examination revealed the alarm pathogen *Streptococcus pyogenes* susceptible to empirical therapy, *Staphylococcus aureus*, *Staphylococcus epidermidis*. The patient was discharged from the clinic on day 9 in good general condition. There were no visual disturbances in the left eye. The only permanent consequence was scarring of the facial skin after surgical access (Fig. 5).

### Case Report 2

**Fig. 6:**
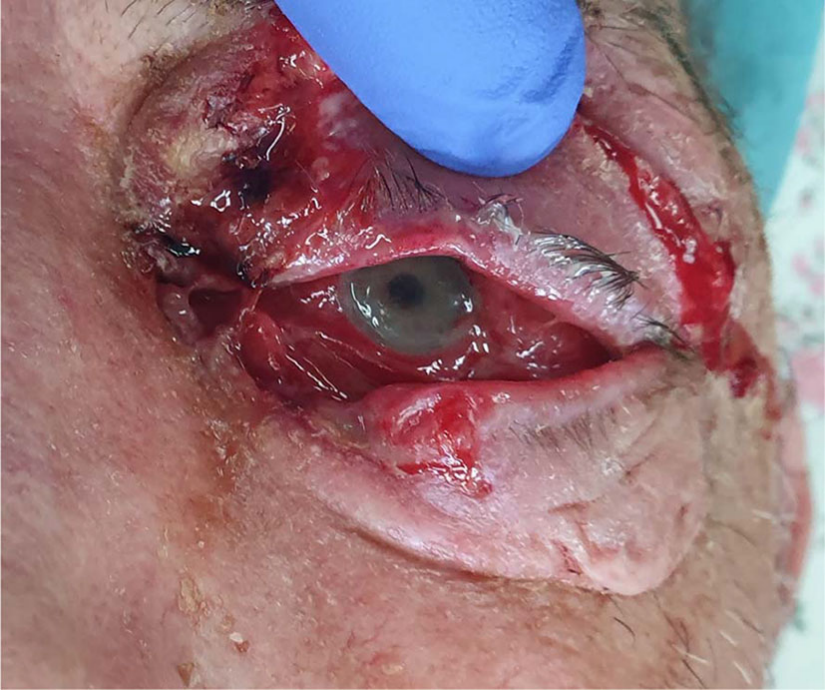
Physical examination shows a contaminated, extensive wound to the skin of the upper eyelid and the left supraorbital area, penetrating the orbital along the roof and the sidewall, from which the exudate of the purulent content emerges. Left eye exophthalmos, the blindness of the left eye, significantly limited mobility of the left eyeball. Due to the swelling, the palpebral fissure was narrowed

**Fig. 7:**
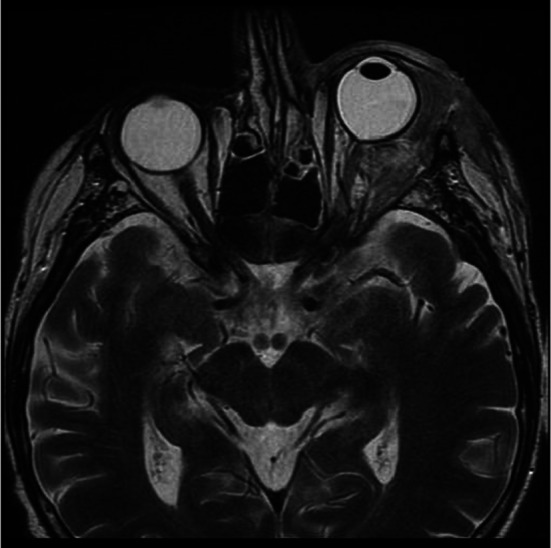
Magnetic resonance imaging orbitals was performed, which showed an image of an abscess of the left orbit, exophthalmos and a forced course of the optic nerve

**Fig. 8:**
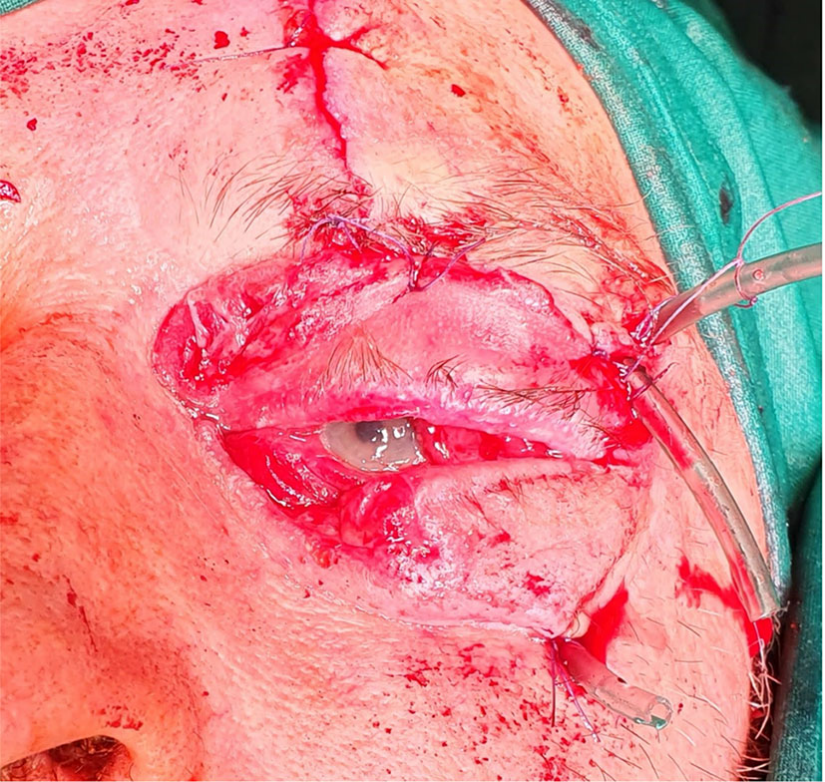
Under general endotracheal anaesthesia, an incision and drainage of the left orbital phlegmon were performed from the supraorbital traumatic wound and suborbital incisions, resulting in abundant purulent exudate. A flow drain was introduced

**Fig. 9:**
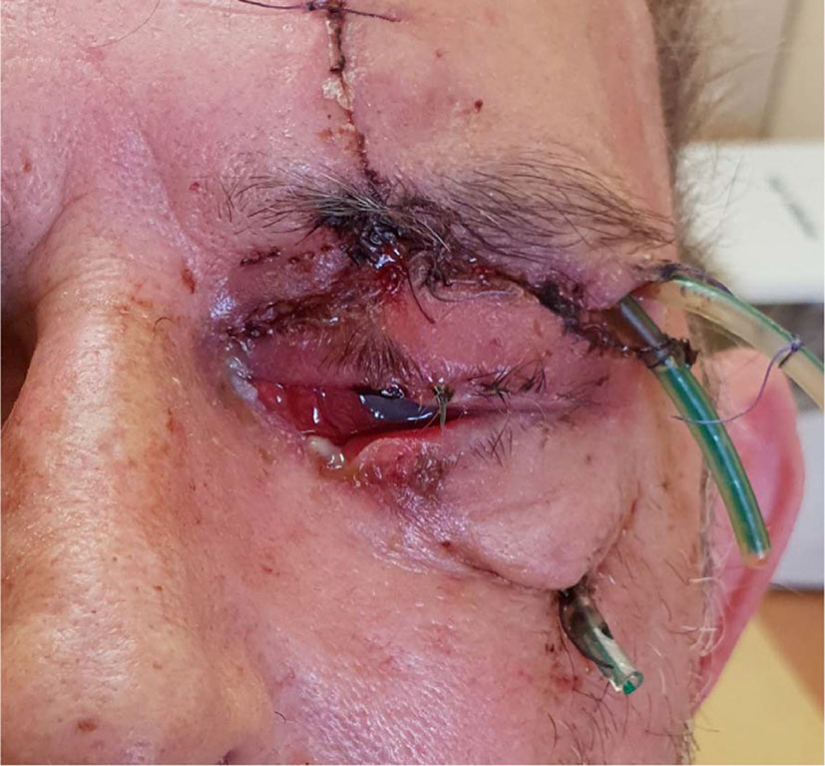
The consequence of the injury and infection was permanent blindness of the left eye

A 63-year-old man was transferred from the department of ophthalmology to the department of maxillofacial surgery due to blindness in the left eye due to orbital phlegmon in order to decompress the abscess. 8 days before hospitalization, the patient suffered a facial injury as a result of hitting a metal gate. Immediately after the injury, the skin wound was treated at the ophthalmology department. Symptoms of acute inflammation appeared on the 5th day after the injury. Physical examination shows a contaminated, extensive wound to the skin of the upper eyelid and the left supraorbital area, penetrating the orbital along the roof and the sidewall, from which the exudate of the purulent content emerges. Left eye exophthalmos, the blindness of the left eye, significantly limited mobility of the left eyeball. Due to the swelling, the palpebral fissure was narrowed. Disturbed sensation in the area of the left orbital. Fracture in the craniofacial skeleton was not detected (Fig. 6). Body temperature was normal. The patient does not report comorbidities and allergies. The patient does not take medications and does not mention any social problems. Magnetic resonance imaging orbitals was performed, which showed an image of an abscess of the left orbit, exophthalmos and a forced course of the optic nerve (Fig. 7). Additionally, a craniofacial CT scan, lung X-ray, ECG were performed, a smear was taken for bacteriological examination and blood was taken for laboratory tests. The patient was administered ceftriaxone 1.2 g intravenous (IV) twice daily, Metronidazole 500 mg three times daily IV, ketoprofen 0.1 g twice daily, enoxaparin 0.4 ml once daily subcutaneous, dexamethasone 8 mg IV once daily. Additionally, drops containing dexamethasone and tobramycin every two hours were used for the left eye. Under general endotracheal anaesthesia, an incision and drainage of the left orbital phlegmon were performed from the supraorbital traumatic wound and suborbital incisions, resulting in abundant purulent exudate. The abscess cavity was rinsed with saline. A flow drain was introduced (Fig. 8). The wounds were surgically prepared and the necrotic masses were removed. In the postoperative period, the level of CRP and WBC was monitored—a decrease in CRP and WBC was observed. A control CT performed on the 3rd day after the procedure showed the correct position of the drain in the eye socket and a significant reduction in exophthalmos. The microbiological examination revealed the alarm pathogen *Streptococcus pyogenes* susceptible to empirical therapy, *Klebsiella pneumoniae*, *Proteus mirabilis*. On the 3rd day after the procedure, the patient reports a subjective sense of light in the left eye (Fig. 9). On the 8th day of hospitalization, the patient was returned to the Ophthalmology Department. The consequence of the injury and infection was permanent blindness of the left eye.

## Discussion

Owing to the retrospective nature of this study, it was granted an exemption by the Poznan University of Medical Science review board.

The first literature case reports of orbital abscess originated in 1884 [5, 6]. However, PubMed research revealed only 254 results using the formula *orbital[title] AND abscess[title]*, and 863 results, when using the formula *orbital[title/abstract] AND abscess[title/abstract]* (and 1359 results for *orbital[all fields] AND abscess[all fields])*.

Orbital abscess formation occur in 8% of patients with retroseptal orbital cellulitis [7].

### Symptoms

The most frequently encountered signs and symptoms include periorbital edema, restricted ocular movement, orbital pain, proptosis, periorbital erythrema, chemosis and vision deterioration—Table 1. [3, 8]–[39]

**Table 1:** Orbital abscess signs and symptoms

Signs and symptoms	Percentage
Periorbital edema	70
Restricted ocular movement	67
Orbital pain	55
Proptosis	55
Periorbital erythrema	45
Chemosis	42
Vision deterioration	39
Purulent discharge	24
Fever	21
Diplopia	18
Facial tenderness	18
Ptosis	15
Face edema	15
Exophtalmos	15
Inability to open an eye	12
An eye mass	9
Vision loss	9
Nausea	6
Facial pain	6
Nasal obstruction	6
Corneal edema	6

Percentage based on current literature review [3, 8]–[39]

### Etiology

Bacterial etiology is the most common and regards pathogenes such as *Streptococcus spp*. [7, 11, 14, 16, 28, 31, 33, 35, 39, 40], *Staphylococcus aureus* [9, 36, 40] (also methycylin resistant *Staphylococcus aureus* [20, 21, 34, 40]) and *Pseudomonas aeruginosa* [24, 30, 41]. Additionally, wide spectrum of bacteria are rarely encountered: *Haemophilus spp*. [28, 39], Coagulase-negative *staphylococcus* [23, 40]*, Peptostreptococcus spp.* [8, 27], *Citrobacter freundii* [11, 40], *Enterobacter spp*. [40], *Enterococcus spp*. [39, 40], *Acinetobacter spp.* [40], *Actinomyces israelii* [40], Diphteroids [40], *Morganella Morgani* [17], *Proteus mirabilis* [17, 40], *Escherichia coli* [40], *Granulicatella Adiacens* [22], *Prevotella melaninogenica* [27], *Eikenella corrodens* [28],, *Propionibacterium acne* [42], *Pseudomonas stutzeri* [38] as well as polymicrobial infections [3, 11, 28, 39, 40]. Gram-negative infections are at higher risk of visual deterioration or loss, especially in regard to Acinetobacter spp. [40] Fungal etiology occurs very infrequently and includes *Exophiala dermatitidis* [15] and *Candida albicans* [11]. Occasionally, the infection etiology remains unknown despite culture sampling and isolation attempt [10, 19, 29, 43]—according to Teena et al. 68.8% of orbit specimens finds the infectious pathogen [40]. Some articles omit stating exact etiology [12, 13, 26].

### Pathogenesis

Orbital abscess formation originates from odontogenic, periorbital, sinonasal, traumatic, or systemic pathologies, like wise iatrogenic complications. Odontogenic pathogenesis includes incorrect or complicated intraoral interventions, such as tooth extractions and endodontic treatment [12, 19, 33, 35] as well as delayed dental procedures related to 'extreme phobia' of dental procedures and severe caries [8, 18]. Common ophthalmological procedures may result in orbital abscess: posterior subtendon injection [9, 15, 29, 34], strabismus surgery [16], trabeculectomy [38], canaliculitis surgical treatment [28], or orbital implants placement [3, 42]. Frequently, the abscess arises from dacryocystitis [17, 23, 27, 37, 44, 45], and rarely from concjuctivitis [20]. Another cause come from sinus pathologies such Pott's Puffy Tumor [13] or frontoethmoidal mucopyocele [30] as well as sinusitis and nonspecific upper respiratory infection [20, 46]. The important origin regards posttraumatic fractures, lacerations and impacted foreign bodies [11, 22, 26]. Finally, systemic conditions such as human immunodeficiency virus (HIV) infection [46], immunosuppression after transplantation [24] or congenital immunodeficiency (in pediatric population) [41]. There are cases where exact pathogenesis remains unknown [25, 32].

### Sequels

Orbital abscess sequels apply not only to the orbit, restricted ocular motility, impaired or lost vision, and central retinal artery occlusion. Infection may spread causing superior orbital fissure syndrome, cavernous sinus thrombosis, meningitis, brain abscess, and subdural empyema [23, 47]–[49]. On the other hand, Hughes et al. reported a case of an orbital abscess concomitant to aseptic meningitis and cavitory lung lesions which pathogenesis concerned severe caries. They claimed hematogenous spread of the infection, because maxillary sinus showed no infection. [8]

### Imaging

Ocular ultrasonography provides immediate assessment of an orbit and opportunity to follow treatment outcomes without unnecessary exposure to radiation [20, 21]. However, more accurate examinations such as CT or MRI are crucial to evaluate local extension and involvement of adjacent structures, especially before surgical treatment. Despite CT is the first line imaging technique in eye infections and pathologies, it has limited power to visualise orbital abscess. In case of severe symptoms and not significant CT examination, additional MRI scans should be performed [21, 25, 50]. According to Sepahdari et al. diffusion-weigted imaging (DWI) of MRI provides accurate imaging of orbital abscess and grants the sufficient tool for patients with renal insufficiency, if used without intravenous contrast. However, they performed a preliminary study with only 9 cases of orbital infections, including 2 lacrimal gland abscess, 2 eyelid abscess, extraconal abscess, intraconal abscess, and subperiosteal abscess [51]. Panoramic radiograph may be used to visualise oral pathologies in case of odontogenic origin of orbital abscess. [31]

### Differential Diagnosis

Numerous conditions present similar symptoms as orbital abscess, possibly misleading the diagnosis, for instance: neoplasms—osteoma of the ethmoid sinus, [52], small cell neuroendocrine carcinoma of the orbit [53] plasmacytoma [54], infections—primary orbital tuberculosis [55], globe subluxation [56], or liquefied hydrogel implant accumulation [57]. On the other hand, physicians reported cases of true orbital abscess primarily misdiagnosed with other pathologies, such as retrobulbar haemorrhage [11], tumor [25], fronto-orbital mucocele, [32] or granulomatosis with polyangitis exacerbation [58]. Therefore, precise diagnostic process is crucial, including past medical history, clinical assessment, imaging, microbiological tests and histopathological evaluation.

### Treatment

According to current review, surgical treatment was necessary in 94% of cases. Abscess drainage is achieved via multiple approaches depending on its localisation: transculuncular, lateral or anterior orbitotomy, Caldwell-Luc approach, intranasal endoscopy, needle aspiration guided by ulstrasound, lower eyelid incision, subcilliar incisio, incision in four quadrants of the orbit. If it is necessary, surgical debridement of necrotic tissues is performed, as well as enucleation or exenteration. Antibiotic therapy is both, initial and supplementary to surgical treatment. Only two cases resolved with alone antibiotics administration—Table 2 [3, 8]–[28, 30, 32]–[39, 42].

**Table 2:** Single case reports of orbital abscess in years 2000–2020

Author	Year	Country	Age	Gender	Etiology	Pathogenesis	Treatment	Results
Iwahashi et al. [15]	2020	Japan	69	Female	*Exophiala dermatitidis*	Complication of subtendon injection	Surgical debridement within two surgeries, antibiotic therapy	Complete recovery
Linton et al. [13]	2019	United Kingdom	16	Male	No stated pathogen	Complication of Pott's tumour	Supraorbital approach, antibiotic therapy	Recovery with persistent mild visual acuity
Wang et al. [14]	2019	China	16	Female	*Streptococcus intermedius*	Complication of sinusitis	Ultrasound-guided drainage, irrigation, antibiotic therapy	Complete recovery
Arora et al. [12]	2018	India	22	Female	No stated pathogen	Complication after tooth extraction by medical fraudster	An incision was given 5 mm below the right lower eyelid, antibiotic therapy	Complete recovery
Rhatigan et al. [9]	2017	Ireland	57	Male	*Staphylococcus aureus*	Complication of posterior subtendon injection	Orbitiotomy via lower lid, antibiotic therapy	Complete recovery
Hughes et al. [8]	2017	Ireland	58	Female	*Peptostreptococcus spp.*	Complication of severe caries	Abscess drainage via lid crease incision, antibiotic therapy	Complete recovery
Procacci et al	2017	Italy	35	Male	Negative microbiological tests		Drainage via subcilliar incision, antibiotic therapy	Complete recovery
Mohammed Saed [11]	2016	United Kingdom	46	Male	*Streptococcus parasinguinis* *Citrobacter freundii* *Candida albicans*	Traumatic craniofacial fractures	Surgical drainage, antibiotic therapy	Recovery with loss of vision in the left eye
Strul et al. [16]	2014	USA	60	Female	*Streptococcus spp.*	Complication of strabismus surgery	Lateral orbitotomy, antibiotic therapy	Recovery with some restriction in abduction
Carruth and Wladis [17]	2012	USA	22	Female	*Proteus mirabilis*	No stated pathogenesis	Orbitotomy with drainage, capsular excision and tarsorraphy, antibiotic therapy	No stated results
					***Two years later***			
					*Morganella morganii*	Complication of dacryocystytis	Transcaruncular orbitotomy with abscess drainage, antibiotic therapy	No stated results
Vijayan et al. [18]	2012		45	Male	*Streptococcus spp.*	Complication of caries	Surgical drainage, antibiotic therapy	No stated results
Kent et al. [3]	2012	Canada	30	Male	Multibacterial infection (gram-positive cocci and rods, gram-negative rods, and anaerobic organisms)	Complication after porous polyethylene implant placement	Surgical removal of the implant and drainage, antibiotic therapy	Recovery with 3 mm residual enophthalmos
De Medeiros et al. [19]	2012	Brazil	No stated age	Female	Negative microbiological tests	Complication after endodontic treatment	Superior medial palpebral technique and inferior palpebral technique, antibiotic therapy	Complete recovery
Secko et al. [20]	2012	USA	36	Female	*Methycylin resistant Staphylococcus aureus*	Complication of conjuctivitis	Surgical drainage, antibiotic therapy	No stated results
Derr and Shah [21]	2012	USA	57	Female	*Methycylin resistant Staphylococcus aureus*	Lower eyelid laceration sequel	Anterior orbitotomy with abscess drainage, antibiotic therapy	No stated results
Teo et al. [22]	2011	Singapore	40	Male	*Granulicatella Adiacens*	Complication of posttraumatic periorbital skin laceration with foreign body	Lateral orbitotomy with abscess drainage and foreign body removal, antibiotic therapy	Recovery with residual proptosis and mild limitation of abduction and adduction
Coskun et al. [23]	2011	Turkey	45	Female	Coagulase-negative *staphylococcus*	Complication of dacryocystytis	Lateral lower lid incision and abscess drainage, antibiotic therapy	Recovery with vision loss
Hull et al. [24]	2011	United Kingdom	65	Male	*Pseudomonas aeruginosa*	Immunosupression after kidney transplantation	Endonasal endoscopic drainage of the abscess, antibiotic therapy	Complete recovery
Qi and He [25]	2010	China	68	Male	*Streptococcus Viridans*	No known direct cause	Drainage of the orbital abscess and debridement of necrotic periorbital soft tissues, antibiotic therapy	Complete recovery
Sousa et al. [26]	2009	Brazil	20	Female	No stated pathogen	Complication of facial trauma	Surgical abscess drainage, antibiotic therapy	Complete recovery
Martins et al. [27]	2009	Brazil	39	Female	*Prevotella melaninogenica* *Peptostreptococcus prevotii*	Complication of dacryocystytis	Subcilliary approach, abscess drainage, antibiotic therapy	Complete recovery
Hatton and Durand [28]	2008	USA	60	Female	*Streptococcus anginosis, Eikenella corrodens, Haemophilus paraphrophilus*	Complication after surgical canaliculitis treatment	medial left upper eyelid crease incision, abscess drainage, antibiotic therapy	Complete recovery
Ram et al. [29]	2008	India	54	Female	Negative microbiological tests	Complication of subtendon injection	Antibiotic therapy	Recovery with adjacent conjunctival and corneal scarring
Kau et al. [30]	2007	Taiwan	74	Male	*Pseudomonas aeruginosa*	Complication of frontoethmoidal mucopyocele	Nasal endoscopic approach, antibiotic therapy	Complete recovery
Hong et al. [42]	2006	Korea	73	Female	*Propionibacterium acne*	Porous Orbital Implant infection	Exenteration, antibiotic therapy	Recovery after exenteration
Kim et al. [31]	2007	Korea	31	Male	*Streptococcus Viridans*	Complication of the periapical abscess of the upper right second and third molars	Antibiotic therapy	Recovery with impaired visual acuity
Aydin et al. [32]	2006	Turkey	77	Female	No pathogen stated	No stated direct cause	Surgical drainage, antibiotic therapy	Complete recovery
Sakkas et al. [33]	2005	Germany	21	Male	*Streptococcus intermedius*	Complication of wisdom tooth extraction	Surgical incision in four quadrants with abscess drainage, antibiotic therapy	Recovery with vision loss
Engelman et al. [34]	2004	USA	90	Female	*Staphylococcus aureus*	Complication of subtendon injection	Fine-needle aspiration of pus, drainage placement, antibiotic therapy	Complete recovery
Zacharaides et al. [35]	2004	Greece	38	Male	*Streptococcus constellatus*	Complication of second maxillary molar extraction	Drainage of abscess via Caldwell-Luc approach, antibiotic therapy	Recovery with vision loss
Irvine and McNab [36]	2002	Australia	80	Female	*Staphylococcus aureus*	Complication after phacoemulsification cataract surgery	Drainage via anterior orbitotomy, antibiotic therapy	Recovery with impaired vision acuity
Ataullah and Sloan [37]	2002	New Zealand	60	Female	*Streptococcus spp.*	Complication of dacryocystytis	Abscess drainage via lower lid incision, antibiotic therapy	Complete recovery
Lebowitz el al. [38]	2001	USA	69	Male	*Pseudomonas stutzeri*	Late complication of trabeculectomy	Enucleation, antibiotic therapy	Recovery after enucleation
Papesch and Philpott [39]	2000	United Kingdom	17	Male	*Haemophilus spp, Streptococcus spp, Enterococcus spp*	Complication of periorbital lacerations	Surgical drainage, antibiotic therapy	Complete recovery

### Outcomes

Complete recovery succeed in 49% of cases, whereas 11% of patients recovered with vision loss, 9% with vision deterioration, 6% with persistent movement restrictions, 3% with exenteration, 3% with enucleation, 3% with residual enophatomos, 3% with residual proptosis, and 3% with corneal scarring. Exact results were not presented in 14% of cases. Fortunately, any patient died in the investigated reports [3, 8]–[28, 30, 32]–[39, 42].( Table 3)

**Table 3:** Studies with more than one case of orbital abscess being reported in years 2000–2020

Author	Year	Country	No cases
Van der Veer [7]	2016	Netherlands	4
Gavriel et al. [59]	2010	Israel	3
Pushker et al. [43]	2009	India	4
Maheshwari et al. [44]	2009	India	6
Eviatar et al. [60]	2009	Israel	3
De Silva et al. [58]	2007	United Kingdom	2
Kikkawa et al. [45]	2002	USA	4
Suneetha et al. [47]	2000	India	13
